# Complete Genome Sequence of Vibrio rotiferianus Strain AM7

**DOI:** 10.1128/MRA.01591-19

**Published:** 2020-05-21

**Authors:** Kentaro Miyazaki, Apirak Wiseschart, Kusol Pootanakit, Kei Kitahara

**Affiliations:** a Department of Life Science and Biotechnology, Bioproduction Research Institute, National Institute of Advanced Industrial Science and Technology (AIST), Tsukuba, Ibaraki, Japan; b Department of Computational Biology and Medical Sciences, Graduate School of Frontier Sciences, The University of Tokyo, Kashiwa, Chiba, Japan; c Institute of Molecular Biosciences, Mahidol University, Salaya, Nakhon Pathom, Thailand; d Ambitious Leader’s Program Fostering Future Leaders to Open New Frontiers in Materials Science (ALP), Hokkaido University, Sapporo, Hokkaido, Japan; e Department of Chemistry, Faculty of Science, Hokkaido University, Sapporo, Hokkaido, Japan; University of Delaware

## Abstract

We isolated the novel strain Vibrio rotiferianus AM7 from the shell of an abalone. In this article, we report the complete genome sequence of this organism, which was obtained by combining Oxford Nanopore long-read and Illumina short-read sequencing data.

## ANNOUNCEMENT

*Vibrio* is a Gram-negative, heterotrophic, and usually aerobic or facultative anaerobic marine bacterial genus belonging to the class *Gammaproteobacteria*. Vibrio rotiferianus was first isolated from cultures of the rotifer Brachionus plicatilis in 2003 (1), and subsequently various species have been isolated from aquatic organisms (2, 3). To date, several genome projects have been initiated for *V. rotiferianus*, but a complete genome sequence has been obtained only for strain B64D1 (4). To gain insight into the genomic evolution of *V. rotiferianus* strains at high resolution, we conducted complete genome sequencing of *V. rotiferianus* strain AM7, which was isolated from an abalone purchased at a fish market in Tokyo, Japan. The shell surface of the abalone was swabbed using a sterilized swab, and single colonies were isolated via streaking onto LB agar plates containing 4% (wt/vol) sea salts (LBSS; Sigma). Eight colonies, which appeared on the plate after incubation at 37°C for 16 h, were investigated via 16S rRNA sequencing analysis of the near full-length of the gene. Seven of the colonies were identified as *Halomonas* spp., and the other was identified as *V. rotiferianus* and designated as AM7. We subjected *V. rotiferianus* AM7 to whole-genome sequencing.

For genomic DNA extraction, AM7 was grown in LBSS broth at 37°C for 18 h. Genomic DNA was prepared using a MagAttract high-molecular-weight (HMW) DNA kit (Qiagen) according to the manufacturer’s instructions. The obtained genomic DNA was subjected to long- and short-read sequencing (5). For long-read sequencing, short genomic fragments were removed using a short-read eliminator (Circulomics). The obtained genomic DNA (1 μg) was used for library construction with a ligation sequencing kit (Oxford Nanopore Technologies [ONT]). The prepared library (without barcoding) was singly applied to a FLO-MIN106 R9.41 flow cell (ONT). Sequencing was performed using a GridION X5 system (ONT). Base calling was performed using Guppy v.3.0.3, generating 148,921 reads (957 Mb) with an average length of 6,426 bases during a 12-h run time (data represent reads obtained after quality [Q] filtering [Q, ≧10; read length, ≧1,000 bases] using NanoFilt v.2.3.0 [6]). The longest read had 181,007 bases.

Short-read sequencing was performed using a MiSeq instrument (Illumina). The library was prepared using a Nextera DNA Flex library prep kit (Illumina), generating paired-end libraries with approximately 350-bp mean insertions. Short-read sequencing was performed using a MiSeq reagent kit v.2 (300 cycles) with 156-bp read lengths. Raw sequencing data were quality trimmed using fastp v.0.20.0 (Q, ≧30; read length, ≧10 bases) (7), yielding 650,801 paired-end reads (100 Mbp) with an average length of 153.3 bp.

For complete *de novo* genome assembly, both long- and short-read data were processed using Unicycler v.0.4.4 (8), followed by a final polishing step using Pilon v.1.23 (9), generating two closed contigs for circular chromosomes and another closed contig for the plasmid. Automatic annotation was then performed using the annotation pipeline DFAST v.1.1.0 (10). The genome statistics and genomic features are summarized in Table 1. Based on the coverage of short reads to the complete chromosome/plasmid sequences, the relative copy number of plasmids to chromosomes was estimated to be approximately 2. The average nucleotide identities to the closest genome sequences were 97.6% between AM7 chromosome 1 and B64D1 chromosome 2 (GenBank accession number CP018312.1) and 96.5% between AM7 chromosome 2 and B64D1 chromosome 1 (accession number CP018311.1).

**TABLE 1:** Genome statistics and genomic features of Vibrio rotiferianus strain AM7

Chromosome or plasmid	Length (bp)	GC content (%)	No. of CDSs^*a*^	No. of rRNAs	No. of tRNAs	Avg read depth (×)	Accession no.
Chromosome 1	3,515,444	44.9	3,062	37	119	30.0	AP019798
Chromosome 2	2,016,094	44.4	1,805	3	15	29.4	AP019799
Plasmid	243,273	42.0	252	0	0	54.1	AP019800

a CDSs, coding sequences.

### Data availability.

The GenBank accession numbers for the complete genome sequence of *V. rotiferianus* AM7 are AP019798 (chromosome 1), AP019799 (chromosome 2), and AP019800 (plasmid) (Table 1). The raw sequencing data were deposited in the DDBJ SRA database under the accession numbers DRR184147 (Illumina data) and DRR184148 (Nanopore data).
